# An analysis of the prevalence and impact of obsessive-compulsive personality disorder on the course of obsessive-compulsive disorder

**DOI:** 10.1192/j.eurpsy.2023.527

**Published:** 2023-07-19

**Authors:** M. Żerdziński, M. Burdzik, P. Dębski

**Affiliations:** ^1^Department of Psychiatry, Center of Psychiatry in Katowice; ^2^Institute of Legal Sciences, University of Silesia, Katowice; ^3^Department of Psychiatry, Faculty of Medical Sciences in Zabrze, Medical University of Silesia in Katowice, Tarnowskie Gory, Poland

## Abstract

**Introduction:**

Obsessive-compulsive disorder (OCD) is a disorder diagnosed on the basis of the presence of obsessions (persistently recurring, intrusive thoughts, images, impulses) and compulsions (compulsive and transiently satisfying actions performed in response to obsessions). A separate clinical phenomenon in the broadly understood anankastic spectrum is obsessive-compulsive disorder (OCPD). OCPD is characterized by scrupulousness, over-normative morality, perfectionism and an inability to make concessions in terms of cooperation. It is assumed that the incidence of OCPD in the course of OCD is 25-32% and it is a report with an unfavorable course and a more difficult therapeutic prognosis. It also means an early onset of OCD with a greater intensity of compulsions and a predominance of symptoms related to the sphere of purity, symmetry and gathering.

**Objectives:**

The main aims of the study were as follows:

1. To assess the prevalence of OCPD in OCD patients.

2. To compare both groups of patients for the severity, level of insight, aggression, impulsiveness and affective symptoms.

3. To verify whether the presence of OCPD depend on factors such as age, gender, treatment duration and delay in starting treatment.

**Methods:**

The study was conducted in a group of 78 patients diagnosed with and treated for OCD. The patients were divided into two groups: patients with OCD and OCPD (group 1, n=43) and patients with OCD without OCPD (group 2, n=35). The groups were subsequently compared for the severity of anankastic symptoms, the level of insight, aggression, impulsiveness and its components and affective symptoms (depression, mania). The following tools were used for the diagnosis: Yale*-*Brown Obsessive Compulsive Scale (Y-BOCS), Hamilton Depression-Rating Scale (HDRS), Young Mania Rating Scale (YMRS), Buss-Perry Aggression Questionnaire (BPAQ), Barratt Impulsiveness Scale (BIS-11), Brown Assessment of Beliefs Scale (BABS) and DSM 5 criteria for OCPD.

**Results:**

Our results confirmed that OCPD is common in OCD and occurred in 55,12% of patients with OCD (n=43; m=21; f=22). The study shows that the presence of OCPD in the course of OCD negatively affects the severity of obsessive-compulsive symptoms, the level of insight, the level of aggression and impulsiveness, and affective disorders. Patients with OCPD obtained higher scores on the YBOCS, BABS, HDRS, and YMRS scales compared to patients with OCD without OCPD. The occurrence of OCPD in the study group did not depend on gender, duration of OCD treatment, or delay in starting it.

**Conclusions:**

The occurrence of OCPD is common in OCD and can cause delay of treatment, worse course, greater intensity of egosyntonic symptoms, and thus worse insight. The presence of OCPD can lead to more hostility and more affective disorders. Screening for OCPD in patients with OCD should be an integral part of an OCD diagnostic process.

**Disclosure of Interest:**

None Declared

